# Is Lung Disease a Risk Factor for Sudden Cardiac Death? A Comparative Case–Control Histopathological Study

**DOI:** 10.3390/diseases13010008

**Published:** 2025-01-06

**Authors:** Ioana Radu, Anca Otilia Farcas, Septimiu Voidazan, Carmen Corina Radu, Klara Brinzaniuc

**Affiliations:** 1 Doctoral School of Medicine and Pharmacy, George Emil Palade University of Medicine, Pharmacy, Science, and Technology of Targu Mures, 540142 Targu Mures, Romania; ioanaradu888@gmail.com; 2 Department of Forensic Medicine Emergency County Hospital, “Constantin Opriș” Baia Mare, 430031 Baia Mare, Romania; 3 Department of Cell Biology, George Emil Palade University of Medicine, Pharmacy, Science, and Technology of Targu Mures, 540139 Targu Mures, Romania; 4 Epidemiology Department, George Emil Palade University of Medicine, Pharmacy, Science, and Technology of Târgu Mureş, 540139 Targu Mures, Romania; septimiu.voidazan@umfst.ro; 5 Institute of Forensic Medicine, 540141 Targu Mures, Romania; carmen.radu@umfst.ro; 6 Department of Forensic Medicine, George Emil Palade University of Medicine, Pharmacy, Science, and Technology of Targu Mures, 540139 Targu Mures, Romania; 7 Department of Anatomy, George Emil Palade University of Medicine, Pharmacy, Science, and Technology of Targu Mures, 540139 Targu Mures, Romania; klara.brinzaniuc@umfst.ro

**Keywords:** sudden cardiac death (SCD), chronic obstructive pulmonary disease (COPD), histopathology, interstitial fibrosis, inflammatory infiltrate, emphysema, lung disease

## Abstract

Background/Objectives: Sudden cardiac death (SCD) constitutes approximately 50% of cardiovascular mortality. Numerous studies have established an interrelation and a strong association between SCD and pulmonary diseases, such as chronic obstructive pulmonary disease (COPD). The aim of this study is to examine the presence of more pronounced cardiopulmonary histopathological changes in individuals who died from SCD compared to the histopathological changes in those who died from violent deaths, in two groups with comparable demographic characteristics, age and sex. Methods: This retrospective case–control study investigated the histopathological changes in cardiac and pulmonary tissues in two cohorts, each comprising 40 cases of SCD and 40 cases of violent death (self-inflicted hanging). Forensic autopsies were conducted at the Maramureș County Forensic Medicine Service, Romania, between 2019 and 2020. Results: The mean ages recorded were 43.88 years (SD 5.49) for the SCD cohort and 41.98 years (SD 8.55) for the control cohort. In the SCD cases, pulmonary parenchyma exhibited inflammatory infiltrate in 57.5% (23), fibrosis in 62.5% (25), blood extravasation in 45% (18), and vascular media thickening in 37.5% (15), compared to the control cohort, where these parameters were extremely low. In myocardial tissue, fibrosis was identified in 47.5% (19) and subendocardial adipose tissue in 22.5% (9) of the control cohort. Conclusions: A close association exists between SCD and the histopathological alterations observed in the pulmonary parenchyma, including inflammation, fibrosis, emphysema, blood extravasation, stasis, intimal lesions, and vascular media thickening in intraparenchymal vessels. Both the histopathological modifications in the pulmonary parenchyma and vessels, as well as those in myocardial tissue, were associated with an increased risk of SCD, ranging from 2.17 times (presence of intimal lesions) to 58.50 times (presence of interstitial and perivascular inflammatory infiltrate in myocardial tissue).

## 1. Introduction

Sudden cardiac death (SCD) has an annual incidence of approximately 4–5 million cases worldwide and approximately 50% of cardiovascular mortality, representing the first cause of death worldwide and a major public health problem [1,2,3]. SCD is defined as a sudden, unexpected death in an apparently healthy patient, with no known cardiac pathological history or within 1 h of the onset of cardiac symptoms [4].

In forensic practice, the most common etiologies of SCD are represented in 80% of cases by CAD (coronary artery disease), followed by cardiomyopathies, coronary hypoplasia, acute myocardial infarction, adiposity cordis, etc. [5].

There are more and more literature data that confirm the association between SCD with chronic obstructive pulmonary disease (COPD), but the pathophysiological mechanism and the interrelationship between them is not fully known [6,7]. The existence of potential determining factors such as chronic systemic inflammation that occurs with advancing age and the development of several chronic diseases, is hypothesized [8,9,10]. Another potential risk factor for the association of these two entities is genetic susceptibility, according to van Suylen et al. [11] there is an association between the DD genotypes of the angiotensin converting gene and the development of right ventricular hypertrophy in individuals with COPD.

After cardiovascular diseases and neoplasias, COPD ranks as the third cause of mortality globally, with a prevalence of about 4–5 million cases per year [12,13]. In Europe, COPD mortality rate varies depending on the country, but in Romania, it exceeds 80 deaths per 100,000 inhabitants [14]. In the US, pulmonary heart disease accounts for 6–7% of all heart diseases in adults and up to 30% of hospitalized heart failure, with the remark that an exact prevalence is difficult to determine because physical examination and routine tests are insensitive for determining pulmonary hypertension and right ventricular dysfunction. The incidence of the pulmonary heart is influenced by environmental factors (pollution) and behavioral factors (smoking, obesity) [15]. COPD is characterized by chronic airways inflammation associated with persistent airflow limitation due to noxious particles and gases [16,17,18].

The histopathological changes induced by COPD in the lungs include small airway remodeling (increased wall thickness, epithelial changes with hyperplasia of muco-secretory cells, and a notable inflammatory infiltrate, especially in the peripheral airways and lung parenchyma, discrete hyperplasia and muscle tunica hypertrophy of the bronchioles) compared to the similar changes observed in the bronchioles of asthmatic patients, as well as fibrosis, a possible pathophysiological mechanism being the production of transforming growth factor—beta (TGF-β), secreted by activated epithelial cells, which subsequently induce local fibrosis [19,20]. Airway inflammation present in COPD is often associated with systemic inflammation, an essential element in maintaining this systemic inflammation being represented by increased levels of TNF alpha. It is well known that increased levels of TNF alpha have a major implication in the production of atherosclerosis, insulin resistance, lipolysis and levels of plasma very-low-density lipoproteins [21,22]. In their study Thomsen M. et al. [23] demonstrated a two- to four-fold increase in the risk of developing cardiovascular disease in patients with systemic inflammation. Additionally, pulmonary blood vessels exhibit changes due to COPD, such as intimal thickening, medial thickening, and plexiform lesions resulting from the activation of endothelial cells and fibroblasts.

The aim of this study is to explore the presence of more pronounced cardiopulmonary histopathological changes in individuals who died from SCD compared to the histopathological cardiopulmonary changes in individuals who died from violent causes, across two cohorts with comparable demographic profiles, age, and sex.

## 2. Materials and Methods

In this retrospective case–control study, the macroscopic and histopathological changes in the heart and lung, organs from 80 forensic autopsies performed in the Maramures County Forensic Medicine Service, Romania, in 2019 and 2020, were analyzed. The 80 cases were divided into 2 groups: group 1, deaths whose cause was sudden cardiac death; and group 2 (control), whose cause of death was violent death.

According to Romanian legislation, all cases of violent deaths, as well as deaths occurring suddenly in apparently good health, without known documented cardiovascular pathologies, which result in death immediately or within one hour of the onset of any symptom in the presence of a witness, or within a maximum of 24 h in their absence, regardless of the place of death or whether resuscitation measures were applied, are classified as medico-legal cases, and a forensic autopsy is mandatory. Thus, the cases of sudden cardiac death were selected on the basis of the history declared by the relatives of the deceased, of the investigations carried out and made available by the criminal investigation bodies, correlating with the autopsy findings.

We excluded from both groups the cases in which the subject’s age exceeded 55 years as it is well known that both cardiovascular and pulmonary diseases show a much higher incidence with advancing age. Additionally, we excluded sudden deaths of non-cardiac origin, deaths in an advanced state of autolysis where histopathological examination would be inconclusive, as well as cases in which toxicological analysis revealed the presence of intoxication due to alcohol or other substances.

This study included 40 consecutive deaths whose cause was SCD (group 1) and control group 2 consisting of 40 cases of violent deaths by self-harm (hanging). Histopathological slides were analyzed, consisting of tissue samples obtained from the heart (specifically, corresponding to the lateral wall of the left ventricle and a fragment from the lateral wall of the right ventricle), each measuring 2 cm in length. For the histopathological examination of the lung, three fragments were taken: one fragment each from the level of the bilateral lower lobes and one fragment from the right middle lobe. The three lung fragments were harvested in one standardized way for all cases, avoiding subpleural harvesting. The interpretation of histopathological changes and their reporting was done globally. Subsequent histopathological processing of the tissue was performed according to the standard protocol following prior fixation in formalin. Histopathological sections were prepared in the usual hematoxylin-eosin staining, and to highlight the fibrosis, stainings were performed to highlight the collagen fibers (Van Gieson and Trichrome Masson staining).

The parameters evaluated at the myocardial tissue level were the following: the presence of epicardial and intracardiac adipose tissue, hypertrophy of cardiac fibers, the presence of necrosis, fibrosis, hypereosinophilia of cardiac fibers, fiber fragmentation, interstitial edema and perivascular and interstitial inflammatory infiltrate. At the lung tissue level, the following parameters were evaluated: inflammation, fibrosis, emphysema, blood extravasation, stasis, intimal lesions at the level of intraparenchymal vessels and, respectively, thickening of the vessel media.

### Statistical Analysis

For the statistical analysis of the data, we used SPSS software version 23, and the Chi-square test was applied, using a threshold of 0.005 for statistical significance. The estimated risk OR interpreted against the-Value of 1 and according to the confidence interval of 95% was also calculated.

## 3. Results

Regarding the demographic characteristics of the two groups, the mean ages were comparable, 43.88 years (SD 5.49) in the case of the group of those who died from SCD, and 41.98 years (SD 8.55) in the case of the control group, without statistically significant differences being recorded (*p* = 0.24). Regarding the gender distribution of the two groups, there were 2 deaths in the group that died from SCD and 5 deaths in the control group, in women (*p* = 0.23).

The histopathological quantification of inflammation within the pulmonary parenchyma was performed using a numerical scale, assigning a score of 0 for the absence of inflammation, a score of 1 for the presence of minimal inflammatory infiltrate, a score of 2 for the presence of moderate inflammatory infiltrate, and a score of 3 for the presence of abundant inflammatory infiltrate. A percentage of 80.0 (32 cases) of the deaths due to violent deaths did not present inflammatory infiltrate at the level of the lung parenchyma (score 0), compared to 42.5% (17 cases) of the deaths caused by SCD. In total, 22.5% (9 cases) of the deaths due to SCD presented an inflammatory infiltrate score of 1, 30.0% (12 cases) presented an inflammation score of 2, and 5% (2 cases) an inflammation score of 3. In the control group, 12.5% (5 cases) had an inflammation score of 1 and 7.5% (3 cases) had an inflammation score of 2; not a single case of score 3 inflammation was recorded in the control group (*p* = 0.002).

For the quantification of fibrosis at the level of the lung parenchyma, staining was performed to highlight the collagen fibers (van Gieson and Masson’s tricorm, respectively). A numerical scale similar to that for the quantification of inflammation was used, giving a score of 0 for the absence of fibrosis, a score of 1 for the presence of fine collagen frames with thicknesses between 5 and 14 microns, and a score of 2 for the presence of collagen bands with thicknesses over 15 microns. Statistically significant differences (*p* = 0.0001) were recorded between the two groups, regarding the microscopic appearance of fibrosis at the level of the lung parenchyma. Thus, 77.5% (31 cases) of the violent deaths did not present fibrosis (score 0), compared to the SCD deaths in which fibrosis was present in a proportion of 62.5% (25 cases), of which the most frequent was fibrosis score of 1 present in 52.5% (21 cases), and 10% (4 cases) showing fibrosis score 2. A percentage of 22.5% (9 cases) of those who died by violent deaths presented a fibrosis score of 1.

**Figure 1:**
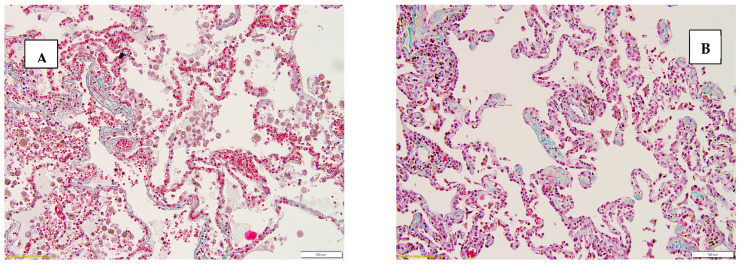

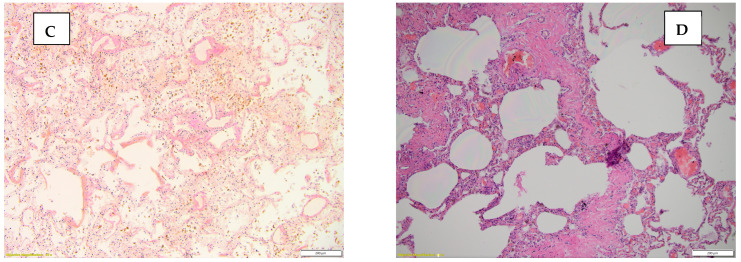
(**A**) Interstitial fibrosis score 2, stained with Masson’s Trichrome, 20×. (**B**) Interstitial fibrosis score 2, stained with Masson’s Trichrome, 20×. (**C**) Interstitial fibrosis score 2, stained with Van Gieson, 10×. (**D**) Interstitial fibrosis score 2 and emphysema, stained with H&E, 40×.

Figure 1 illustrates sections of pulmonary parenchyma stained with Van Gieson and Masson’s Trichrome staining techniques.

In both groups, comparable percentages were recorded for emphysema, 85.0% (34 cases) of deaths in the control group where this lesion, albeit in a focal manner, while 92.5% (37 cases) of the deaths due to SCD presented emphysema, but over more extensive areas of the lung on the histopathologically examined pulmonary parenchyma (*p* = 0.48).

Another evaluated parameter within the pulmonary parenchyma was the presence of intra-alveolar hematic extravasation, which was quantified with a score of 2 for the presence of abundant hematic extravasation, a score of 1 for the presence of a reduced amount of blood extravasation and a score of 0 for the absence of blood extravasation. The presence of intra-alveolar blood extravasation was found more frequently in the case of deaths due to SCD, 37.5% (15 cases) with a score of 1 and 7.5% (3 cases) with a score of 2, compared to 2.5% (1 case) with a score of 2 in the control group, these differences being statistically significant (*p* = 0.01).

The vascular changes in the pulmonary parenchyma from violent deaths in the control group were more subtle compared to the group of SCD deaths. Consequently, intimal lesions were absent in the control group, whereas in the group of deaths due to SCD, 15% (6 cases) exhibited either ballooning of the endothelium or focal denudation of the endothelium, with statistically significant differences observed (*p* = 0.01). The mean increase was less often found in the control group 10% (4 cases) compared to 37.5% (15 cases) in the case of SCD, and in this case, the difference was statistically significant (*p* = 0.008) (Table 1).

Organ fibrosis contributes to approximately 45% of all deaths recorded worldwide, with the lungs and heart being the most frequently involved organs in fibrosis-related fatalities [24]. Hinderer S. et al. [25] state that ischemic heart disease and endomyocardial fibrosis constitute the main etiology of end-stage heart failure. Myocardial interstitial fibrosis arises due to a disturbance in the extracellular matrix turnover, leading to impaired function and structure of the entire heart [26,27]. There are studies such as that of Nauffal V. et al. [28] that prove the association between myocardial interstitial fibrosis and the occurrence of disorders of rhythm such as atrial fibrillation or heart failure, through this mechanism SCD may occur.

During the histopathological examination of the myocardial tissue, a numerical scale was used to quantify the degree of fibrosis, giving a score of 0 for the absence of fibrosis, a score of 1 for fine collagen frames with thicknesses between 5 and 15 microns, a score of 2 for the presence of thin bands of collagen with thicknesses between 16 and 30 microns and score 3 in the case of the presence of thick bands of collagen, more than 30 microns thick. Thus, extremely statistically significant results were revealed between the two groups (*p* = 0.0001). In 100% of the deaths due to SCD, the presence of fibrosis with a score ≥1 was highlighted, compared to 52.5% (21 cases) of the control group in which fibrosis was not evident. What is worrying, however, is the fact that compared to the mean age of the investigated cases, which is young, of this active population, 37.5% (15 cases) of the control group had fibrosis in the form of fine frames (score 1) and 10% (4 cases) more advanced degrees of fibrosis. A relevant aspect highlighted in our study is represented by the association of SCD in 60% (24 cases) with fibrosis score 2, followed by fibrosis score 3 in 27.5% (11 cases).

**Figure 2:**
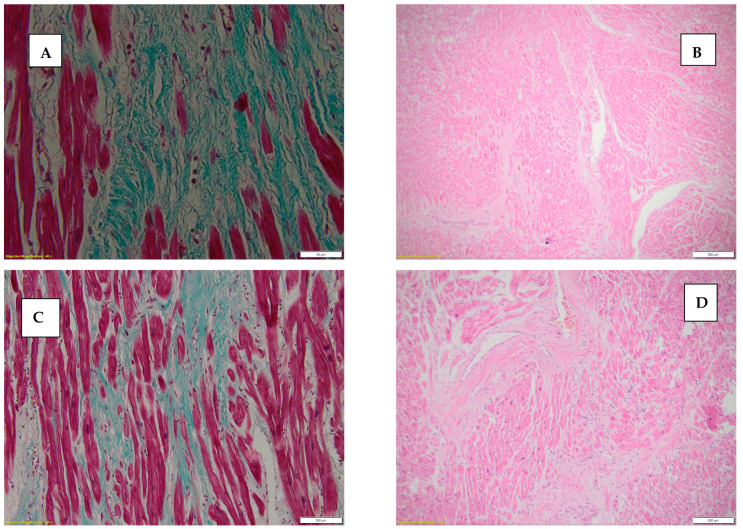
(**A**) Myocardial interstitial fibrosis score 2, stained with hematoxylin-eosin, 4×. (**B**) Myocardial interstitial fibrosis score 3, stained with Masson’s Trichrome, 20×. (**C**) Interstitial fibrosis score 3, stained with Van Gieson, 40×. (**D**) Interstitial fibrosis score 2 with interstitial inflammatory infiltrate, stained with hematoxylin-eosin, 10×.

Figure 2 depicts interstitial myocardial fibrosis, stained with hematoxylin-eosin and Masson’s Trichrome.

The histopathological changes present in early myocardial ischemia are obvious in hypereosinophilia of cardiomyocytes, undulation of cardiac myocardial fibers, interstitial edema, nuclear changes and fragmentation of contractile bands [29]. Hypereosinophilia of cardiomyocytes present in the case of deaths from SCD was found in 72.5% (29 cases) compared to the control group, in which it occurred in only 5% (2 cases); similar results are also found in the case of fiber hypertrophy, these being present in the case of group 1 at 75% (30 cases) and 7.5% (3 cases) in the control group, the results being statistically significant, *p* = 0.0001, in both cases (Table 2).

Another histopathological change associated with prolonged myocardial ischemia is cardiac tissue necrosis, a parameter also evaluated in our study. This was observed in 12.5% of SCD cases; however, it was absent in cardiac sections from the control group (*p* = 0.02).

**Figure 3:**
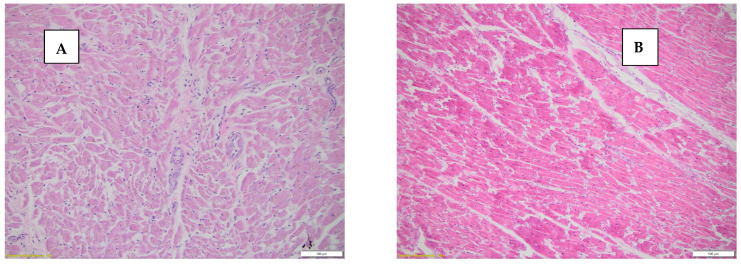
(**A**) Myocardial tissue with fragmented cardiac fibers, HE stain, 20×. (**B**) Myocardial tissue with intact, unfragmented fibers, HE stain, 20×.

Although in the case of deaths by SCD, fiber fragmentation presents a characteristic histopathological change produced by acute myocardial ischemia, it was present in 57.5% (23 cases), but in the case of violent deaths, 5% (2 cases) presented myocardial fiber fragmentation (Figure 3).

In the case of the inflammatory infiltrate evaluated at the cardiac tissue level, the results were extremely conclusive: in the case of violent deaths, 97.5% (39 cases) did not present a pathological inflammatory infiltrate compared to those who died from SCD, in which more than half (24 cases) presented an inflammatory infiltrate, predominantly localized perivascularly, but an interstitial inflammatory infiltrate was also evident.

**Figure 4:**
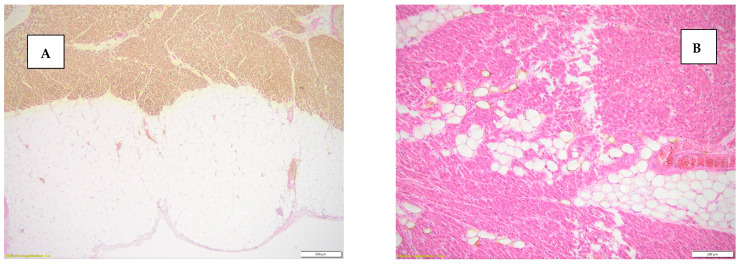
(**A**) Excessive epicardial adipose tissue, van Gieson stain, 4×. (**B**) Intramyocardial adipose tissue (adipositas cordis), hematoxylin-eosin stain, 10×.

It is known that the presence of myocardial adipose tissue is strongly associated with the risk of sudden death in the young and middle-aged population. The results of our study highlighted the fact that an extremely significant proportion of the control group 22.5% (9 cases) presented excessive epicardial adipose tissue, pathological epicardial adipose tissue was also found in 52.5% (21 cases) of the death group recorded by SCD (Figure 4).

## 4. Discussion

Cardiac interstitial edema consists of the accumulation of fluid in the interstitial space that leads to the impairment of cardiac function influencing both contractility and relaxation [30,31]. It is well known that there is a marked variety of pathological conditions including hypertension, hypoproteinemia, pulmonary hypertension, myocardial infarction, and coronary sinus hypertension that can lead to the presence of interstitial edema at the level of the heart [31,32]. In our study, interstitial edema was highlighted in an extremely significant proportion at 87.5% (35 cases) in those from group 1 compared to those from the control group, where it was present only in 25% (10 cases), (*p* = 0.0001).

In normal myocardial tissue, a small number of inflammatory cells such as macrophages, lymphocytes and, rarely, mast cells can be seen, but the presence of neutrophils and monocytes always draws attention to a pathological process [33,34]. The presence of inflammatory infiltrate in the heart can be associated with viral, bacterial or fungal myocarditis, poisoning with various substances or drugs, immune-mediated systemic diseases, ischemia and reperfusion injuries or associated with the presence of epicardial adipose tissue [35,36,37]. It can be composed of neutrophils, lymphocytes, macrophages, and eosinophils.

Packer M. [37] highlights that in systemic inflammatory diseases, pathological adipogenesis occurs at the epicardial level, accompanied by an excessive accumulation of epicardial adipose tissue and an overproduction of pro-inflammatory adipokines, which eventually lead to cardiac fibrosis.

The normal thickness of the right ventricular wall is up to 5 mm, and a variable amount of adipose tissue can be visualized normally subepicardially, this being located antero-laterally and apically. If this amount of adipose tissue is extensive, it is called adipose infiltration and must be differentiated from arrhythmogenic right ventricular dysplasia (ARVD), which consists of the replacement of myocardial tissue at the level of the right ventricle with fibro-adipose tissue, leading to the appearance of ventricular arrhythmias, thus increasing the risk of SCD [38,39].

In COPD, the epithelial cells at the level of the lung parenchyma secrete inflammatory mediators such as TNFα, IL-1, IL-6, reactive oxygen species and proteases that lead to the maintenance of chronic inflammation at this level. The inflammatory infiltrate in COPD is mainly composed of macrophages, neutrophils, and T lymphocytes [40]. This inflammation, however, is not a localized one, limited only to the lung parenchyma and airways, but is a systemic inflammation that is associated with an increased risk to develop cardiovascular disease or lung cancer [41,42,43,44]. In our study, we demonstrated that the presence of inflammation in the pulmonary parenchyma increases the risk of SCD by 5.41× (OR = 5.41 (CI95% = 1.99–14.66)).

The pathogenic mechanisms responsible for the production of emphysema act in several ways: CD8+ T lymphocytes secrete proteases such as Granzyme B, which lead to the degradation of the extracellular matrix. Furthermore, proteases secreted by neutrophils and alveolar macrophages lead to the degradation of connective tissue and elastin at the alveolar level, with emphysema arising as a consequence of these proteolytic actions. Another pathogenic mechanism involved in emphysema is the impairment of endothelial cell function by products secreted from inflammatory cells, smooth muscle cells, and fibroblasts [6]. There are numerous studies, among which we mention those of Voelkel NF et al. [45] and Rabe KF et al. [46], that conclude that there is an interrelationship between pulmonary hyperinflation from COPD (transposed microscopically by the presence of emphysema) and cardiovascular diseases, a possible pathophysiological mechanism being represented by the damage to the pulmonary vessels, microcirculation alterations and degradation of endothelial cells. The results obtained in our study reveal a 2.17× increase in the risk of death from SCD in the case of the association of pulmonary emphysema.

The presence of interstitial fibrosis at the level of the lung parenchyma was associated with a risk of death by SCD of 5.74× (CI95% = 2.15–15.29). Over half (52.5%), that is 21 cases from group 1, simultaneously presented the association of fibrosis score ≥ 1 both at lung and heart level, this association being present in only 10% (4 cases) of the control group. A possible explanation for this association found so frequently in those who died from SCD can be represented by the fact that systemic inflammation produces the activation of myofibroblasts with the release of growth factors and cytokines, including increased amounts of transforming growth factor-β1 (TGF-β1) and WNT1-inducible signaling protein-1 (WISP1) which plays an extremely important role in the fibrosis process [47,48]. However, the activation of cardiac myofibroblasts plays a key role in maintaining the integrity of the myocardium and tissue repair after myocardial infarction. An excessively active maintenance of myofibroblasts leads to a decrease in tissue compliance, the amount of oxygen and nutrients, cardiomyocytes becoming atrophic. The consequence of these pathological processes is represented by the onset of left ventricular dysfunction [49].

Of the cases of deaths as a result of self-harm included in this study, 85% did not present intra-alveolar blood extravasation, compared to 55% of the deaths by SCD. The presence of intra-alveolar hemorrhage is associated with a 4.63× risk (OR 4.63; CI95% = 1.59–13.49) of dying from SCD. Intra-alveolar hemorrhages occur due to the destruction of the basal membrane of the alveolar capillaries (alveolar–capillary basement membrane) and can be caused by extremely varied conditions and pathologies such as: sepsis, vasculitis, systemic autoimmune diseases, and Goodpasture’s syndrome [50], viral infections (influenza virus, SARS, dengue fever, Nile virus fever) or bacterial (tuberculosis), pulmonary hypertension, poisoning (herbicides and pesticides), medication administration (cytostatics [51], sevoflurane [52]). However, in order to accurately establish the mechanism and interrelationship between SCD and the presence of intra-alveolar hematic extravasation, additional investigations and research are needed.

It is well known that obstructive pulmonary diseases affect not only the airways but also the lung vasculature, with histopathological changes frequently observed in the pulmonary vessels. These changes include intimal thickening and arteriolar muscularization, with the subsequent loss of capillaries and precapillary arterioles. Another pathological change in the vessels encountered in COPD is represented by the presence of in situ thrombosis, vascular congestion and stasis. All these changes are often accompanied by the presence of perivascular inflammatory infiltrate [53]. In our study, the pulmonary vascular changes encountered in the group of deaths by SCD were more marked compared to the control group: in 15% (6 cases) of those who died by self-harm, stasis was absent, compared to 2.5% (1 case) of the group of deaths by SCD. The presence of stasis is associated with a 6.88× higher risk of death from SCD than in the absence of stasis (OR 6.88; CI95% = 0.789–60.06). Also, the presence of intimal lesions was associated with a 2.17× higher risk of death by SCD (OR 2.17; CI95% = 1.70–2.78), as well as the thickening of the vascular media presents a 5.40× higher risk of death by SCD (OR 5.40; CI 95% = 1.60–18.20).

The histopathological changes evident in the heart such as hypereosinophilia of myocardial fibers, their fragmentation, the presence of interstitial edema, interstitial fibrosis, perivascular and interstitial inflammatory infiltrate as well as the presence of necrosis, may be due to multiple etiological factors and are signs of an acute or chronic damage to the myocardium [54]. Arrhythmias, coronary thrombosis, pulmonary thromboembolism, congestive heart failure, and sepsis are among the most common causes that produce these histopathological changes corresponding to acute incipient or prolonged ischemia, all of which are associated with an increased risk of death from SCD. The results of our study revealed that the presence of some histopathological changes in the myocardial tissue were associated with a very high risk of death from SCD. Thus, hypereosinophilia of cardiomyocytes is associated with a 50.09× higher risk of death from SCD compared to cases in which this histopathological change is absent (OR 50.091; CI 95% = 10.294–243.736). Cardiomyocyte fragmentation was associated with a 25.70× higher risk of death from SCD, compared to those without cardiomyocyte fragmentation (OR 25.70; CI 95% = 5.43–121.59). Also, the presence of interstitial edema increases the risk of death by SCD by 21.0× in those in whom it is present compared to those in whom it is absent (OR 21.00; CI 95% = 6.45–68.27). One of the most worrying parameters that was associated with an extremely high risk of death by SCD was the presence of perivascular and interstitial inflammatory infiltrate, increasing the risk of death by SCD by 58.50× (OR 58.50; CI 95% = 7.28–469.80). Cardiac fiber hypertrophy also carries a 37.00× higher risk of death from SCD when present compared to its absence (OR 37.00: CI 95% = 9.33–146.65). A lower risk of death from SCD was associated with the presence of subepicardial adipose tissue, which increased by 3.80× of SCD (OR 3.80; CI 95% = 1.44–10.01).

Considering the risk of death from SCD associated with histopathological changes both at the level of the heart and at the level of the lung parenchyma, it is necessary to implement solid measures to prevent CAD, which is the most frequent cause of death from SCD, as well as the implementation of some screening programs for patients with high risk of death from SCD. Their monitoring to identify possible episodes of early myocardial ischemia by dosing new markers that have proven their usefulness such as Cx43, JunB, and TUNEL assays could represent a turning point in the prevention of SCD [55]. Also, the implementation of screening programs and careful monitoring of patients with lung diseases is necessary to reduce the number of sudden cardiac deaths.

The limitations of this study are represented by the relatively small groups originating from a single geographical area, the population of this area being able to present pathologies associated with the effects of pollution specific to this area. Another limitation is the limited period of time in which the study took place, as well as the absence of information related to risk factors such as smoking.

## 5. Conclusions

Sudden cardiac death represents approximately 50% of cardiovascular mortality and the first cause of death worldwide. There is an interrelationship and a close association between SCD and the histopathological changes present in the lung parenchyma such as inflammation, fibrosis, emphysema, stasis, intimal lesions at the level of intraparenchymal vessels and, respectively, thickening of the vessel media. Both the cytopathological changes in the parenchyma and pulmonary vessels as well as those in the myocardial tissue were associated with an increased risk of death from SCD between 2.17× (presence of intimal lesions) and 58.50× (presence of interstitial and perivascular inflammatory infiltrate at myocardial tissue level). In order to decrease the risk of death from SCD and to prevent the causes of sudden cardiac death, screening programs are needed to evaluate patients not only from a cardiological point of view, but also to identify risk factors such as smoking and pollution. It is also necessary to identify early lung diseases that have an association and a close interrelationship with SCD.

## Figures and Tables

**Table 1:** A comprehensive overview of the parameters assessed within the pulmonary parenchyma.

Group	Inflammation				*p*-Value	Fibrosis			*p*-Value	Hematic Extravasation			*p*-Value	Intimal Vascular Lesions		*p*-V alu e	Vascular Wall Thickening		Emphysema	
	Score 0	Score 1	Score 2	Score 3		Score 0	Score 1	Score 2		Score 0	Score 1	Score 2		Score 0	Score 1		Score 0	Score 1	Score 0	Score1
Group 1 (SCD)	42.5% (17)	22.5% (9)	30.0% (12)	5.0% (2)	*p* = 0.002	37.5% (15)	52.5% (21)	10% (4)	*p* = 0.0001	55.0% (22)	37.5% (15)	7.5% (3)	*p* = 0.01	85.0% (34)	15% (6)	*p* = 0.01	62.5% (25)	37.5% (15)	7.5% (3)	92.5% (37)
Group 2 (control)	80.0% (32)	12.5% (5)	7.5% (3)	0.0% (0)		77.5% (31)	22.5% (9)	0% (0)		85.0% (34)	12.5% (5)	2.5% (1)		100% (40)	0% (0)		90% (36)	10% (4)	15% (6)	85% (34)

**Table 2:** Details of the parameters assessed at the cardiac level.

Group	Fibrosis				*p*-Value	Hypereosinophilia		*p*-Value	Fiber Fragmentation		*p*-Value	Fiber Hypertrophy		*p*-Value
	Score 0	Score 1	Score 2	Score 3		Score 0	Score 1		Score 0	Score 1		Score 0	Score 1	
Group 1 (SCD)	0% (0)	12.5% (5)	60% (24)	27.5% (11)	*p* = 0.0001	27.5% (11)	72.5% (29)	*p* = 0.001	42.5% (17)	57.5% (23)	*p* = 0.0001	25% (10)	75% (30)	*p* = 0.001
Group 2 (control)	52.5% (21)	37.5% (15)	7.5% (3)	2.5% (1)		95% (38)	5% (2)		95% (38)	5% (2)		92.5% (37)	7.5% (3)	
**Group**	**Interstitial Edema**				***p*-Value**	**Perivascular Inflammatory Infiltrate**		***p*-Value**	**Subepicardial Adipose Tissue**		***p*-Value**	**Necrosis**		***p*-Value**
	**Score 0**		**Score 1**			**Score 0**	**Score 1**		**Score 0**	**Score 1**		**Score 0**	**Score 1**	
Group 1 (SCD)	12.5% (5)		87.5% (35)		*p* = 0.0001	40% (16)	60% (24)	*p* = 0.0001	47.5% (19)	52.5% (70)	*p* = 0.001	87.5% (35)	12.5% (5)	*p* = 0.02
Group 2 (control)	70% (30)		25% (10)			97.5% (39)	2.5% (1)		77.5% (31)	22.5% (9)		100% (40)	0.0% (0)	

## Data Availability

The original contributions presented in this study are included in the article; further inquiries can be directed to the corresponding authors. The data presented in this study are available on request from the corresponding author due to privacy, legal reasons, and ethical reasons.
